# 1393. Unveiling disease trends: a ten-year analysis of mandatory notification diseases in Brazil (2013-2022)

**DOI:** 10.1093/ofid/ofad500.1230

**Published:** 2023-11-27

**Authors:** Beatriz Barraqui Nardo, Marco Aurélio Fagundes Angelo, Débora de Vasconcelos, Naísses Zóia Lima, Walisson Ferreira Carvalho, Ana Paula Ladeira, Braulio Couto

**Affiliations:** UNIFENAS - Universidade José do Rosário Vellano, Alfenas, Minas Gerais, Brazil; Hospital Risoleta Tolentino Neves (HRTN), Belo Horizonte, Minas Gerais, Brazil; Hospital Risoleta Tolentino Neves (HRTN), Belo Horizonte, Minas Gerais, Brazil; PUC MInas, Belo Horizonte, Minas Gerais, Brazil; PUC MInas, Belo Horizonte, Minas Gerais, Brazil; Biobyte Tecnologia em Epidemiologia, Belo Horizonte, Minas Gerais, Brazil; Biobyte Tecnologia em Epidemiologia, Belo Horizonte, Minas Gerais, Brazil

## Abstract

**Background:**

Disease surveillance is crucial for timely detection and control of diseases. In Brazil, mandatory disease notification is required through the National System of Notifiable Diseases (SINAN). This paper aims to contribute to the understanding of disease burden and patterns in Brazil, a representative region of South America and other developing countries.

**Methods:**

The study utilized a retrospective observational study design to conduct surveillance of 54 mandatory notifiable diseases over a 10-year period, from January 2013 to December 2022. The data was collected from a reference public hospital located in Belo Horizonte, a city with a population of approximately 3,000,000 inhabitants, located in the Minas Gerais State of Brazil. Monthly incidence rates were calculated for each disease by using the number of cases as the numerator and the total number of patients admitted to the hospital as the denominator. To estimate endemic levels, the 90th percentile was used for both the monthly incidence rate and the mobile yearly average rate, which provided a threshold for identifying unusually high disease incidence levels. Secular time trends were evaluated using linear regression analysis.

**Results:**

Over 10 years, 37,081 notifiable diseases were diagnosed from 54 different diseases. Only five diseases accounted for 87% of cases: classical dengue fever, unspecified communicable disease, assault by unspecified means, toxic effect of unspecified substance, and work-related conditions (Fig. 1). Monthly endemic levels were estimated, revealing an increase in incidence for 15 diseases including HIV, Syphilis, and assault by unspecified means (Fig. 2). Analysis of pandemic phases showed consistent increase in diseases such as visceral leishmaniasis and tuberculosis between 2018 and 2022 (Fig. 3), emphasizing the need for continued vigilance and targeted interventions.

**Figure 1:**
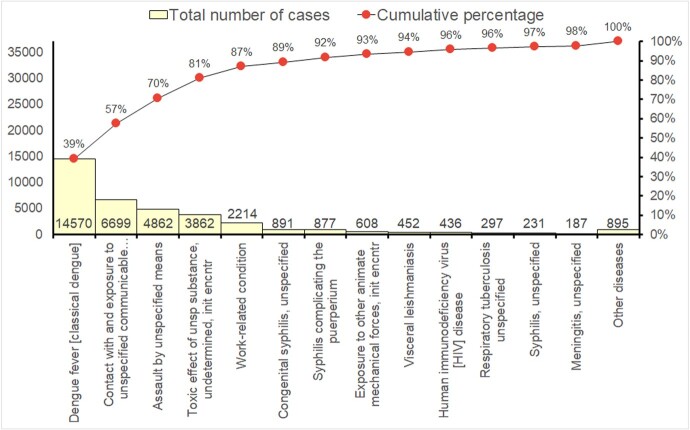
Pareto Chart illustrating the occurrence of 54 notifiable diseases over a 10-year period (2013-2022) in a reference public hospital in Belo Horizonte, Brazil.

**Figure 2:**
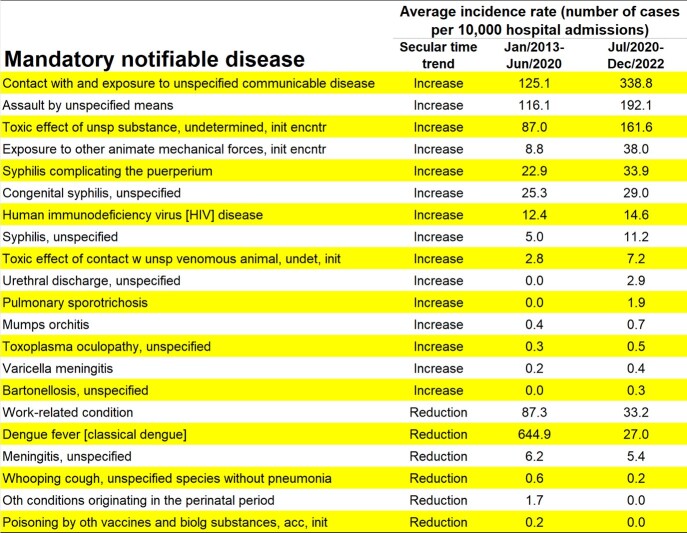
Trend analysis of notifiable diseases with incidence changes over a 10-year period (2013-2022) in a reference public hospital in Belo Horizonte, Brazil.

**Figure 3:**
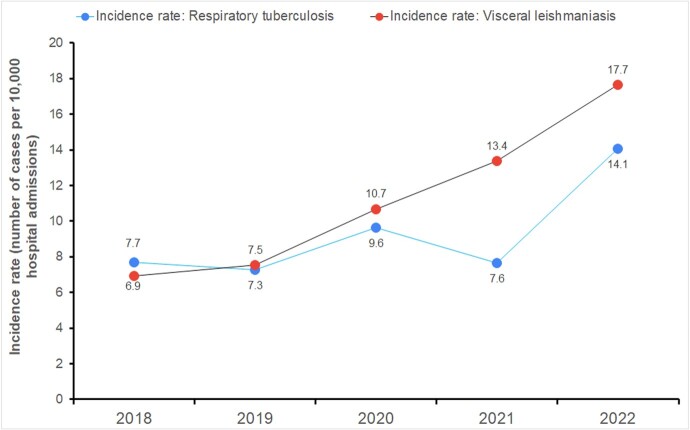
Incidence rate of visceral leishmaniasis and respiratory tuberculosis during pre-pandemic, pandemic, and late-phase pandemic periods (2018-2022) in a reference public hospital in Belo Horizonte, Brazil.

**Conclusion:**

This study provides valuable insights into the epidemiology and trends of 54 mandatory notifiable diseases in Belo Horizonte, Brazil, over a 10-year period. The findings of this study contribute to our understanding of the burden of these diseases in the study population and may inform public health strategies for disease prevention and control in the future.

**Disclosures:**

**All Authors**: No reported disclosures

